# CYLD and SUMO in neuroblastoma therapy

**DOI:** 10.18632/oncoscience.287

**Published:** 2016-01-29

**Authors:** Katarzyna Chmielarska Masoumi, Ramin Massoumi

**Affiliations:** Department of Laboratory Medicine, Translational Cancer Research, Division of Molecular Tumour Pathology, Lund University, Sweden

**Keywords:** CYLD, SUMO, SENP, neuroblastoma

Cylindromatosis gene, CYLD is a tumor suppressor gene that was originally identified as a gene mutated in familial cylindromatosis and related diseases [1]. CYLD belongs to the deubiquitination enzymes family, which function by removing of ubiquitin chains from different proteins. The ubiquitin-specific protease domain of CYLD is located at the C-terminus and mutations in this region inactivate the capacity of CYLD to cleave polyubiquitin chains from specific substrates. CYLD contains three cytoskeleton-associated protein-glycine-conserved (CAP-Gly) in its amino terminus [1]. The CAP-Gly domains consists of about 70 amino acid residues important for the re-localisation of CYLD to the perinuclear region or as a scaffold for substrates [5]. CYLD negatively regulates the NFκB pathway by deubiquitinating important factors such as NFκB essential modulator (NEMO, also known as IκB kinase γ), TRAF2 (TNF Receptor-Associated Factor 2) and TRAF6 (TNF Receptor-Associated Factor 6). Beside NFκB, CYLD has been show to modulate other signaling pathways such as Wnt, MAPK, and Bcl-3. Regulation of these signaling pathways are important for the tumor suppressor function of CYLD in different types of human cancer [5].

Recently, we have demonstrated a significant role of CYLD in differentiation of neuroblastoma [3]. Neuroblastoma is the most common extracranial tumor of childhood and it is originating from primordial neural crest cells that give rise to sympathetic neural ganglia and adrenal medulla. The age of patients, genetic aberrations, and metastatic spread are important factors with regard to treatment decisions and prognosis. Surgery, chemotherapy, radiotherapy, and autologous stem cell transplantation are commonly used as treatment therapy for neuroblastoma patients. In addition, forced differentiation of neuroblastoma cells by retinoids treatment is used mostly as a therapy for high-risk neuroblastomas. Treatment with retinoids leads to elimination of residual neuroblastoma cells that survive after chemotherapy or stem cell transplantation [6, 7].

**Figure 1:**
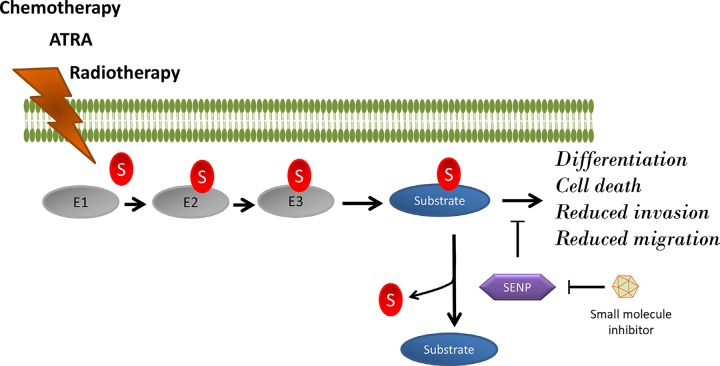
SUMOylation of any substrate is directed by the action of SUMO-activating (E1), SUMO-conjugation (E2), and SUMO-ligation enzymes (E3) This process is reversed by SUMO specific proteases (SENPs). A combination therapy including traditional chemotherapy, radiotherapy, and/or ATRA together with SENP inhibitors can elevate the levels of SUMOylated protein in cancer cells that in turn promotes cell differentiation, cell death, and reduced migration/invasion of neuroblastoma cells.

Recent work from our laboratory, demonstrated that all-trans retinoic acid (ATRA)-induced differentiation of neuroblastoma cells. It, operates by increasing in the expression of the small ubiquitin-like modifier (SUMO) protein that can affect downstream signaling inside the cells by quickly switch on or off different cellular processes, including survival, proliferation, and differentiation. The SUMOylation is reversible through the action of SUMO specific proteases (SENPs) (Figure 1). We observed that a short ATRA treatment of neuroblastoma causes a transient SUMOylation of CYLD that reduces its deubiquitin activity, as well as activation of NF-κB signaling via ubiquitination of TRAF2/TRAF6. However, prolonged ATRA treatment reduced CYLD SUMOylation and promoted cell death instead. Therefore, it was proposed that the balance between non-SUMOylated and SUMOylated CYLD can direct neuroblastoma cancer cells against differentiation or cell death via regulation of NF-κB signaling [3]. Furthermore, high CYLD expression is associated with better overall and relapse-free survival and inversely correlated with the stage of neuroblastoma [3].

In this context, maintaining high expression levels of CYLD and SUMO proteins together can be used promising tools for future therapeutic strategies for neuroblastoma patients. Indeed, cellular death induced by oxidative stress via overexpression of SUMO-1 was previously shown to be blocked by the activation of deSUMOylated enzyme SENP1 [2]. Beside cell death, inhibition of SENP1 reduces migration and invasion of cancer cells [8]. Thus, inhibitors against SENP can be a novel anticancer tool for treating neuroblastoma. Recently, a number of small molecule inhibitors against SENP isoforms have been developed and tested using *in-vitro* approaches [4]. Future studies need to exploit the therapeutic potential of SENP inhibitors in combination with chemotherapy, radiotherapy, and ATRA treatment for neuroblastoma.
